# 

**Published:** 1883-07

**Authors:** 


### STATE CONVENTION OF VETERINARY SURGEONS IN CHICAGO.

We noticed in the May number of the *United States Veterinary Journal* a call for a State convention of veterinary surgeons at Chicago, in order to adopt measures calculated to improve and protect the welfare of the profession. Prominent among the signers we notice the name of our friend, Dr. W. Sheppard, of Ottawa, a gentlemen of acknowledged ability and very enthusiastic in all that appertains to veterinary science. The Doctor was elected First Vice-President. He is a very active worker, and gave ample evidence of it, not only in directing and perfecting the organization, but in reading an essay on lameness, in order to keep the ball rolling. We congratulate the veterinary profession of Illinois for the step taken in advancing the interest and welfare of veterinary science, and in its choice of able workers. May the good work extend.—*Spirit of the Times.*

### BOOKS AND PAMPHLETS RECEIVED.

L. HIPPOPHAGIE ET LES VIANDES INSALUBRES par M. E. Decroix. Paris.

This pamphlet gives the history and progress of the use of horse flesh as food and proves that while the meat obtained from the horse, mule and ass is not so tender as other meats it is equally nutritious and more economical. In Paris hippophagie is on the increase, 10,000 horse and 500 mules and asses being consumed in one year. It is an advantage to the proprietor, causes improvement in the stock and is endorsed by the Society for the Prevention of Cruelty to Animals.

"DEMODEX THYLLOIDES" IN THE SKIN OF CANADIAN SWINE. By R. R. Wright, of Toronto. Reprint from proceedings, Canadian Institute.

Mr. Wright states that this cutaneous parasite exists to some extent in the pork sent to the Toronto market. Its occurrence hitherto has been recorded only by Dr. Csokor, of the Veterinary Institute of Vienna. The parasite causes sub-cutaneous abscesses, the size of a hazel nut. As it is confined to the skin and does not affect the general health of the animal, the treatment consists simply in the removal of the parts involved, thereby preventing the flesh from becoming unfit for food.

TRICHINÆ. By W. C. W. Glazier, M. D., Detroit. Johns Hopkins University Circulars, Annals et Bulletin de la Société de Medicine de Gand.

JOURNALS.—La Clinica Veterinaria Milano.

This number occupies itself principally in recording the favorable results of Anthrax Vaccination according to Pasteur's method as practised in Turin and other places in Italy and on the diagnosis of rabies with communications from Pasteur, Roux, Chamberland and Thuillier; also one from Paul Bert on the same disease.

The Veterinarian London. The Veterinary Journal, London. Der Zoologische Garten, Frankfort. Schweizerisches Archiv fur Theirheilkunde, und Thierzucht, Bern. Archiv fur Wissenschaftliche und Practische Thierheilkunde, Berlin. Journal de la Société contre L'Abus du Tabac, Paris. Kansas City Review of Science and Industry. Chicago Medical Journal and Examiner. The Medical Record. New York. The College and Clinical Record, Philadelphia. Annals of Anatomy and Surgery, Brooklyn. New England Medical Monthly, Sandy Hook. Chicago Medical Review. Virginia Medical Monthly, Richmond. Nashville Journal of Medicine and Surgery. The Southern Clinic, Richmond. The Western Medical Reporter, Chicago. Journal of Cutaneous and Venereal Diseases, New York. Denver Medical Times. The St. Joseph Medical Herald. San Francisco Western Lancet. The Weekly Medical Review, Chicago and St. Louis. The Blacksmith and Wheelwright, New York. National Live Stock Journal, Chicago. The Breeder's Gazette, Chicago. Cultivator and Country Gentleman, Albany. Indiana Farmer, Indianapolis. Truth, San Francisco. Weekly Drover's Journal, Chicago. The Chicago Tribune. The Medical Register, Philadelphia. Spirit of the Times, New York. United States Veterinary Journal, Chicago. Horseshoer and Hardware Journal, Chicago.

Tidskrift for Veterinar-Medicinc och Husdjursskotsel utgifven af C. A. Lindqvist, Stockholm. Giornale di Anatomia, Fisiologia e Patologia, degli Animali, Pisa. Il Medico Veterinario, Torino.

